# Reciprocal Regulation of NF-kB (Relish) and Subolesin in the Tick Vector, *Ixodes scapularis*

**DOI:** 10.1371/journal.pone.0065915

**Published:** 2013-06-12

**Authors:** Victoria Naranjo, Nieves Ayllón, José M. Pérez de la Lastra, Ruth C. Galindo, Katherine M. Kocan, Edmour F. Blouin, Ruchira Mitra, Pilar Alberdi, Margarita Villar, José de la Fuente

**Affiliations:** 1 Department of Veterinary Pathobiology, Center for Veterinary Health Sciences, Oklahoma State University, Stillwater, Oklahoma, United States of America; 2 SaBio, Instituto de Investigación en Recursos Cinegéticos IREC-CSIC-UCLM-JCCM, Ciudad Real, Spain; Swedish University of Agricultural Sciences, Sweden

## Abstract

**Background:**

Tick Subolesin and its ortholog in insects and vertebrates, Akirin, have been suggested to play a role in the immune response through regulation of nuclear factor-kappa B (NF-kB)-dependent and independent gene expression via interaction with intermediate proteins that interact with NF-kB and other regulatory proteins, bind DNA or remodel chromatin to regulate gene expression. The objective of this study was to characterize the structure and regulation of *subolesin* in *Ixodes scapularis. I. scapularis* is a vector of emerging pathogens such as *Borrelia burgdorferi, Anaplasma phagocytophilum* and *Babesia microti* that cause in humans Lyme disease, anaplasmosis and babesiosis, respectively. The genome of *I. scapularis* was recently sequenced, and this tick serves as a model organism for the study of vector-host-pathogen interactions. However, basic biological questions such as gene organization and regulation are largely unknown in ticks and other arthropod vectors.

**Principal Findings:**

The results presented here provide evidence that *subolesin*/*akirin* are evolutionarily conserved at several levels (primary sequence, gene organization and function), thus supporting their crucial biological function in metazoans. These results showed that NF-kB (Relish) is involved in the regulation of *subolesin* expression in ticks, suggesting that as in other organisms, different NF-kB integral subunits and/or unknown interacting proteins regulate the specificity of the NF-kB-mediated gene expression. These results suggested a regulatory network involving cross-regulation between NF-kB (Relish) and Subolesin and Subolesin auto-regulation with possible implications in tick immune response to bacterial infection.

**Significance:**

These results advance our understanding of gene organization and regulation in *I. scapularis* and have important implications for arthropod vectors genetics and immunology highlighting the possible role of NF-kB and Subolesin/Akirin in vector-pathogen interactions and for designing new strategies for the control of vector infestations and pathogen transmission.

## Introduction

Subolesin, initially called 4D8, was discovered as a tick protective antigen in a mouse model of *Ixodes scapularis* (Acari: Ixodidae) infestations [1]. Subolesin gene and protein sequences are highly conserved in ticks and are the ortholog of insect and vertebrate Akirins [2]–[6]. RNA interference (RNAi) studies demonstrated that *subolesin* knockdown profoundly affects tick biological processes such as feeding and fertility and resulted in degeneration of tick guts, salivary glands, reproductive tissues and embryos [2]–[4], [7]–[9]. In subsequent studies, Subolesin was found to control tick gene expression, impact the innate immune response, and affect tick infection by *Anaplasma phagocytophilum, A. marginale*, *Babesia bigemina* and *Borrelia burgdorferi* [2]–[4], [7]–[9], [10]–[18]. Additionally, vaccination with Subolesin/Akirin demonstrated promising results for the control of arthropod vector infestations and pathogen infection/transmission [16]–[18].

The evolution of *subolesin*/*akirin* has been characterized in metazoans but little is known about gene organization and function in ticks [2]–[6], [13], [17]. Furthermore, although functional studies have been conducted in several species including ticks, little is known about *subolesin*/*akirin* gene regulation [2]–[5], [13], [17]. Subolesin/Akirin have been suggested to play a role in the immune response through regulation of nuclear factor-kappa B (NF-kB)-dependent and independent gene expression through interaction with intermediate proteins that interact with NF-kB and other regulatory proteins, bind DNA or remodel chromatin to regulate gene expression [3], [5], [13], [17]–[21]. However, further experiments are required to characterize the role of Subolesin/Akirin in the immune response.

Recently, *subolesin* and *NF-kB* (*Relish*) gene knockdown in ticks suggested that NF-kB might participate in the regulation of *subolesin* expression while Subolesin may be involved in the regulation of *NF-kB* (*Relish*) expression [3]. The NF-kB transcription factor is a key molecule in various cellular processes such as proliferation, cell death, development and innate and adaptive immunity [19]. These results suggested the hypothesis that NF-kB (Relish) may play a role in the regulation of *subolesin* expression, thus adding an additional complexity to the immune response in ticks.

The objective of this study was to characterize the structure and regulation of *subolesin* in the tick vector, *I. scapularis*. *I. scapularis* is a three-host tick with a telotropic host behaviour that transmits emerging pathogens such as *B. burgdorferi, A. phagocytophilum* and *Babesia microti* that cause human Lyme disease, anaplasmosis and babesiosis, respectively [22]–[25]. *I. scapularis* is a model organism for the study of vector-host-pathogen interactions [26], [27]. Recent developments in *I. scapularis* genomics, proteomics and functional genomics have advanced our understanding of tick biological processes [26], [27]–[30]. However, basic biological aspects such as gene organization and regulation are largely unknown in ticks. The information obtained from these studies advances our understanding of gene organization and regulation in *I. scapularis* and other tick species.

## Results and Discussion

### The *subolesin* Gene Sequence and Structure are Highly Conserved

**Figure 1:**
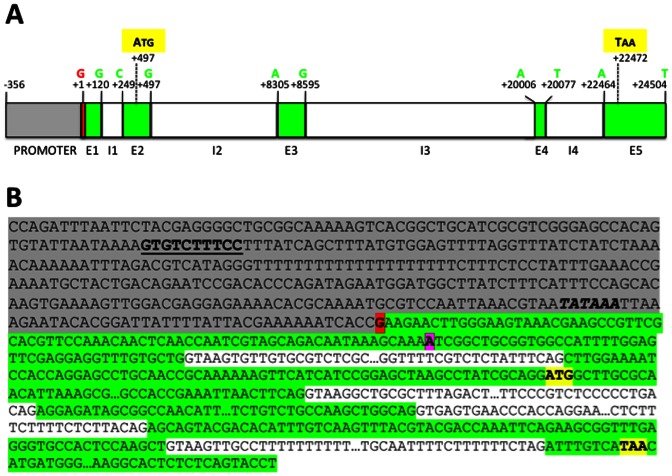
*subolesin* gene organization and sequence. (A) *subolesin* gene organization is shown containing promoter (grey), exon (E; green) and intron (I; white) regions. Major transcription start site at position +1 is shown in red. The first and last positions for exon sequences are shown in green. Translation start and stop codons are shown in yellow with positions marked for the first base of the codon. (B) *subolesin* gene sequence is shown using the same colors depicted in (A). The core promoter is shown with putative NF-kB binding site (bold underlined), TATA box (bold italics), major (red) and minor (purple) transcription start sites indicated. Translation start and stop codons are shown in yellow. Only exon/intron junction sequences are shown. In the mRNA, polyA starts after the last nucleotide in exon 5.

Five exons and four introns were identified in the *subolesin* gene. From 5′ to 3′, exons 1–5 had 120 bp, 249 bp, 291 bp, 72 bp and 2,041 bp, respectively, and introns 1–4 had 128 bp, 7,807 bp, 11,410 bp and 2,386 bp, respectively (Figs. 1A and 1B). The alignment of *subolesin* gene sequences identified a total of 186 candidate polymorphisms with 5 single-nucleotide polymorphisms (SNPs) and 3 insertions/deletions (INDELs) on exon sequences and 156 SNPs and 22 INDELs located on intron sequences (Table 1). These polymorphisms and particularly INDELs could be attributed to errors in the assembly of the *I. scapularis* genome and/or to inter-strain variations that are common in ticks [31]–[33]. Additionally, as in most *akirin* genes [6], the last tick *subolesin* exon was made mostly of untranslated sequences (Figs. 1A and 1B).

**Table 1:** Characterization of polymorphisms in *subolesin* exon and intron sequences.

EXON/INTRON	SNPs	INDELs
Exon 1	0	0
Intron 1	1	0
Exon 2	2	0
Intron 2	60	7
Exon 3	4	0
Intron 3	59	7
Exon 4	0	0
Intron 4	36	8
Exon 5	0	1
Total	161	25

The number of SNPs and INDELs was recorded after *subolesin* sequence alignments performed between Wikel tick colony and ISE6 tick cells genomic sequences for introns and between Wikel tick colony genomic sequence and ISE6 tick cells and tick strains cDNAs for exons.

**Figure 2:**
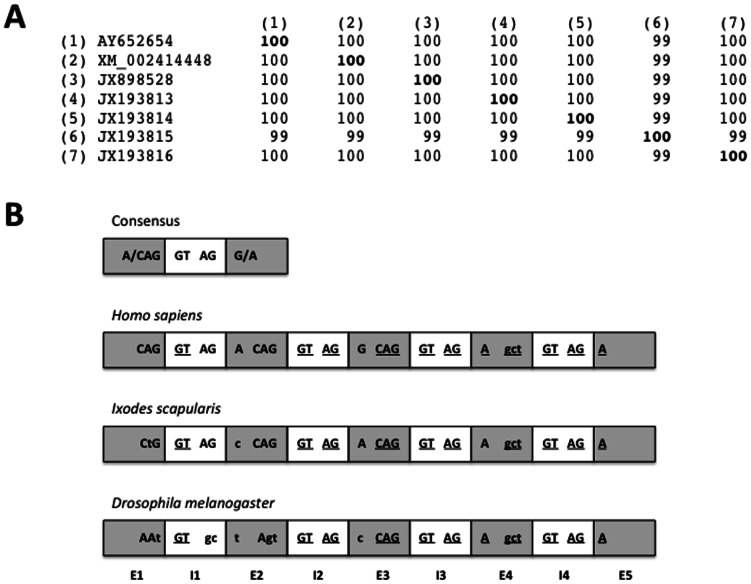
*subolesin* gene sequence and structure are highly conserved. (A) Percent identity matrix created by Clustal 2.1 for *subolesin* coding sequences in *I. scapularis* tick cell lines (1, IDE8; 3, ISE6) and tick strains (2, Wikel; 4, OSU; 5, NY; 6, RI1; 7, RI2). (B) Exon/intron/exon junction sequences in *subolesin* and *akirin* genes. The exon/intron/exon junction sequences are highly conserved in all organisms (consensus sequence). Human (*akirin2*; AC_000138), tick (JX898528) and fly (AE014296) *subolesin*/*akirin* gene exon/intron/exon junction sequences are shown. Nucleotides that differ from consensus sequence are shown in lower case letters. Nucleotides conserved among all three sequences are underlined. Abbreviations: E, exon; I, intron.

The *subolesin*/*akirin* sequences are highly conserved in ticks and other invertebrates and vertebrates [2]–[6]. Coding sequence identity at the nucleotide level was greater than 99% for all tick sequence pairwise comparisons (Fig. 2A). As reported by Macqueen and Johnston [6], our results support the conservation of gene organization (5 exons and 4 introns) in tick *subolesin* and insect and vertebrate *akirins* (Fig. 2B).

The exon/intron/exon junction sequences are highly conserved in all organisms with a consensus (<exon><GT–intron–AG><exon>) sequence [34] (Fig. 2B). In tick *subolesin*, all intron sequences at the <exon><intron><exon> junctions had the consensus sequence (Fig. 1B), which was conserved in human, tick and *Drosophila melanogaster* fly intron 2–4 sequences (Fig. 2B). Although tick <exon><intron> or <intron><exon> junction sequences diverged from consensus in exons 1, 2 and 4, these polymorphisms were common in other organisms (Fig. 2B). In general, *subolesin*/*akirin* <exon><intron><exon> junction sequences were conserved between different organisms and highly similar to the consensus (Fig. 2B). Remarkably, <exon 4><intron 4> junction sequence diverged from consensus but was conserved in human, tick and fly genes (Fig. 2B).

### The *subolesin* Core Promoter Region is Located within 356 Nucleotides Upstream of the Transcription Start Site

Two putative transcription start sites were identified in tick *subolesin* (Fig. 1B). The strongest peak after mRNA start site analysis by automated capillary electrophoresis suggested that the major transcription start site was located at a guanine, 316 bases before the translation start site (Fig. 1B). The rapid amplification of cDNA ends (5′-RACE) analysis of *subolesin* cDNA corroborated both major and minor transcription start sites in 60% and 20% of sequenced clones, respectively, suggesting the possibility of more than one transcript for *subolesin* gene in tick cells.

**Figure 3:**
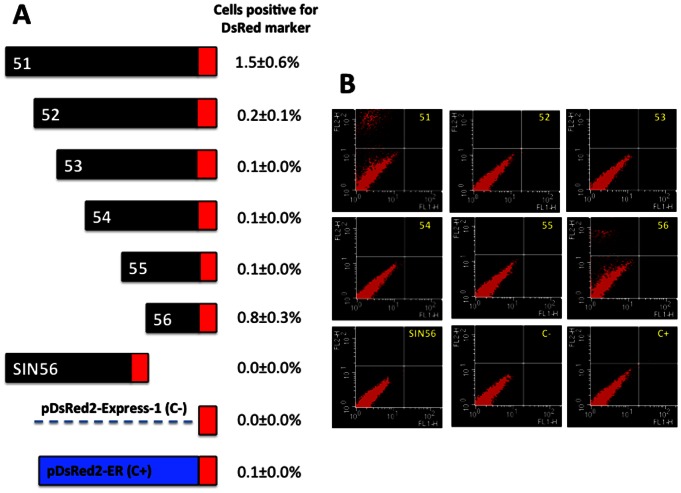
The *subolesin* core promoter is located in a fragment 356 bp upstream of the major transcription start site. (A) *subolesin* promoter fragments tested in ISE6 tick cells and percent positive cells for DsRed fluorescent marker (Ave±S.D.; N = 2). (B) Flow cytometry profile of transiently transfected and control cells 24 h after transfection.

**Figure 4:**
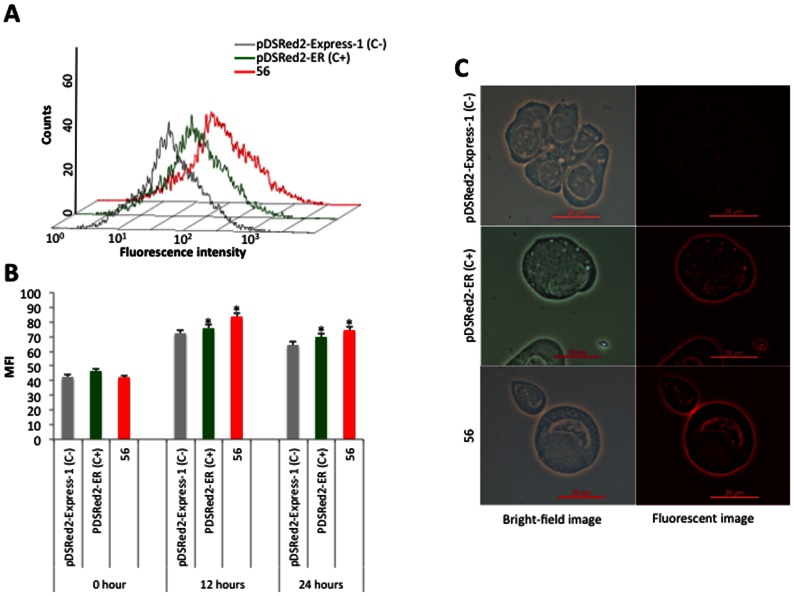
The *subolesin* core promoter is active in mosquito cells. (A) Flow cytometry profile histogram of mosquito Aag2 cells 12 h after transient transfection with pDsRed2-ER expressing the DsRed protein (positive control; C+), promoterless pDsRed-Express-1 (negative control; C-) and the construct containing the identified *subolesin* core promoter (fragment 56). (B) Geometric median fluorescence intensity (MFI) in transiently transfected Aag2 cells 0, 12 and 24 h after transfection. MFI values (Average±S.D.; N = 2) were compared between C+ and fragment 56 transfected cells and C- by Student’s T-test (*P≤0.05). (C) Representative images of imunofluorescence analysis of transiently transfected Aag2 cells 12 h after transfection. Bars, 20 µm. Two independent experiments were conducted with similar results.

Seven fragments (51–56 and SIN56) of the *subolesin* 5,503 pb promoter region were amplified by PCR, cloned and sequenced (Fig. 3A). The 51 to 56 fragments shared the same 3′end with increasing deletions at the 5′ end, while the SIN56 fragment had the same 5′ end as the full-length 5,503 bp fragment 51, but with a 769 bp deletion at the 3′ end (Fig. 3A and Table 2). These promoter fragments were characterized for their ability to direct the expression of DsRed fluorescent marker in ISE6 tick cells. Although transfection efficiency of ISE6 tick cells was very low (approximately 0.1%; C+ in Figs. 3A and 3B), FACS analysis of transfected tick cells was consistent in two different experiments and showed that between 0.1±0.0% and 1.5±0.6% of the cells were positive for the DsRed marker in all constructs but SIN56 (Figs. 3A and 3B). These results suggested that the core promoter region [35] necessary and sufficient for *subolesin* expression in tick cells was located on the 56 fragment (Figs. 3A and 3B), containing 356 bp upstream of the major transcription start site (Fig. 1B). To further confirm these results, the reporter construct containing the 56 fragment with the *subolesin* core promoter region was transfected into mosquito Aag2 cells in which transfection efficiency was higher than in tick cells (35–40% transfected cells). The results confirmed the activity of the identified *subolesin* core promoter region in Aag2 cells (Figs. 4A–4C). In the *subolesin* core promoter region, a putative TATA box and NF-kB-binding sites were predicted at positions −41 to −46 and −279 to −270 with respect to the major transcription start site, respectively (Fig. 1B).

**Table 2:** Primers and PCR conditions used for *subolesin* intron amplification.

Intron	Upstream/downstream primer sequence (5′-3′)	PCR conditions (annealing, extension)
INTRON 1	56: TCAGCCATTTCAAAGTTGCG 3PR: GCTCCGGATGATGAACTTTT	56°C, 30 s 72°C, 1 min
INTRON 2	INT23: CAAATCAACCCTTCACCCTTC INT231: TATCTGTAGGGTGCAACGCA INT252: ACAGTGAAATGCATCGGCTT INT25: ACTCCAGGGGAGACGAGAA	52°C, 30 s 72°C, 4 min 56°C, 30 s 72°C, 4 min
INTRON 3	INT33: ATGATGAAGGAGCGCGAGA INT331: TTCGGTGCAGTTCCCTATTA INT351: AAAAGTGGAAAGAGGAGGGA INT35: CCTCAAACCGCTTCTGAATT	56°C, 30 s 72°C, 5 min 30 s 56°C, 30 s 72°C, 3 min
INTRON 4	INT45: CCAAATTCAGAAGCGGTTTG INT43: CCACCATGGGTTTCTTCTTT	56°C, 30 s 72°C, 3 min

### NF-kB Transcription Factors are Present in ISE6 Tick Cell Nuclear Extracts

**Figure 5:**
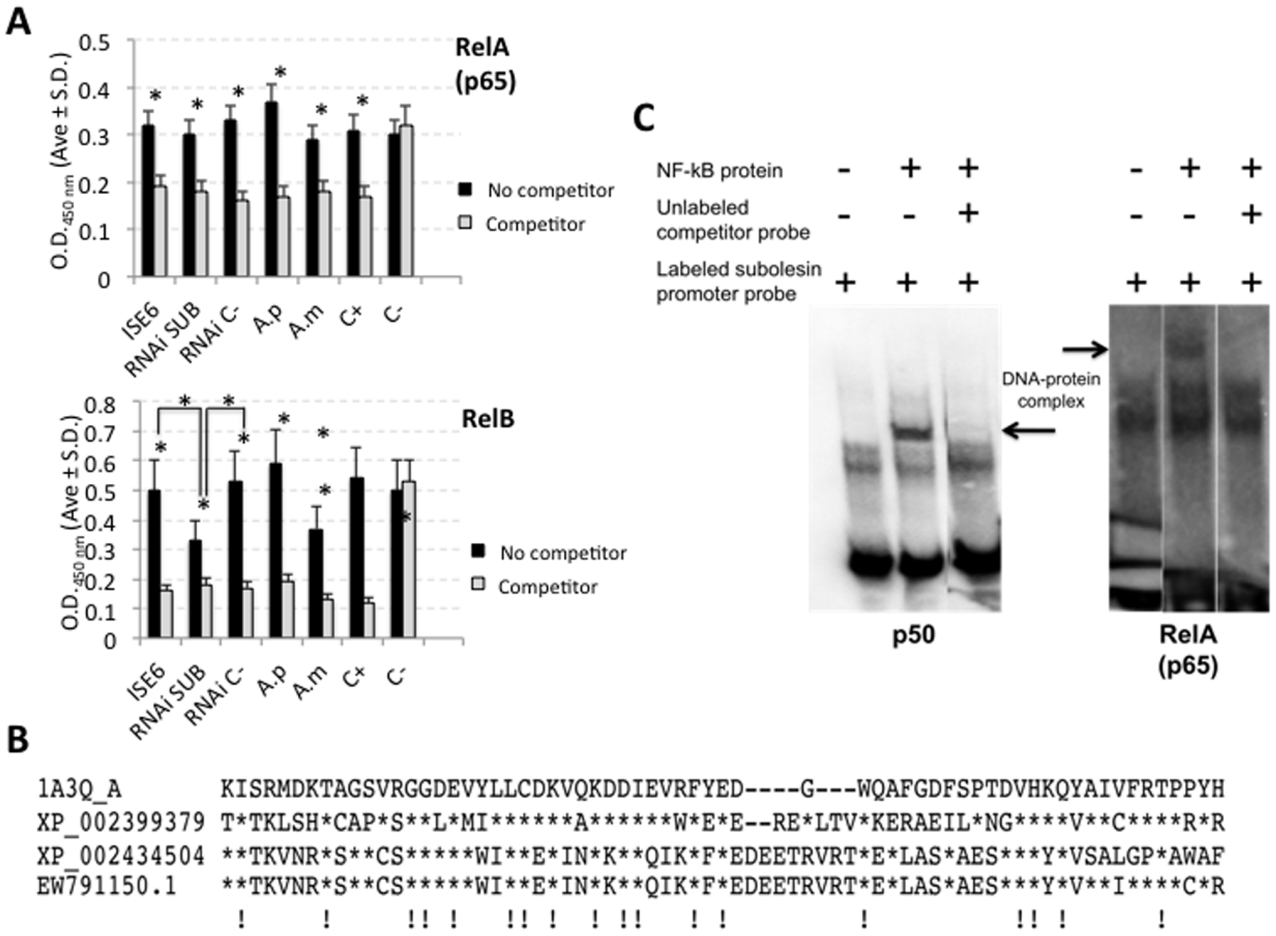
NF-kB factors are present in *I. scapularis* tick cells and bind to *subolesin* promoter. (A) NF-kB-binding activity in ISE6 tick cells nuclear extracts. A DNA-binding ELISA was used with RelA (p65) and RelB antibodies to characterize NF-kB-binding activity in ISE6 tick cells. Nuclear extracts were prepared and assayed from untreated ISE6 tick cells (ISE6), tick cells after incubation with *subolesin* dsRNA (RNAi SUB) or the unrelated *Rs86* dsRNA (RNAi C-), and *A. phagocytophilum* (A.p) and *A. marginale* (A.m) infected tick cells. The wild-type consensus NF-kB-binding oligonucleotide was used as a competitor for NF-kB binding in order to monitor the specificity of the assay when compared to non-competing conditions. The mutated consensus oligonucleotide which should have no effect on NF-kB binding was used as negative control (C-) while Raji cells nuclear extracts were used as positive control for NF-kB binding (C+). Results (Ave±S.D.; N = 4) were compared between groups by Student’s T-test (*P≤0.05). (B) Protein sequence alignment of the IPT-NF-kB domain (cd01177) in putative *I. scapularis* NF-kB factors. Asterisks denote identity to human reference sequence (1A3Q_A). Amino acids conserved in all sequences are shown with (!) at the bottom of the alignment. (C) NF-kB transcription factor binds to *subolesin* promoter. Representative electrophoretic mobility shift assay showing *subolesin* promoter-NF-kB interactions. Labeled dsDNA probe corresponding to the *subolesin* promoter was incubated with p50 and RelA (p65) NF-kB transcription factors. The unlabeled probe used to compete DNA-protein interactions corresponded to the predicted NF-kB-binding site in the *subolesin* promoter (GTGTCTTTCC). Fast migrating unbound probes are found at the bottom of the gel whereas protein–DNA complexes have slower mobility (arrows).

NF-kB (RelA (p65) and RelB)-binding activity was characterized in nuclear extracts of ISE6 tick cells under different treatments (Fig. 5A). The results demonstrated the presence of RelA and RelB-like factors in tick cell nuclear extracts (Fig. 5A). Competition with the consensus NF-kB-binding site oligonucleotide suggested that binding activity in tick cells extracts was specific and similar to that obtained with Raji cell nuclear extracts (Fig. 5A). As in previous experiments with *Relish* [3], *subolesin* knockdown resulted in the inhibition of RelB but not RelA binding activity (Fig. 5A), probably reflecting a reduction in tick NF-kB protein levels.

The search for NF-kB-like sequences in *I. scapularis* databases resulted in three sequences (XP_002399379, XP_002434504, EW791150; the last sequence is likely an EST derived from XP_002434504) with homology to NF-kB transcription factors (Fig. 5B). All these sequences contained the IPT-NF-kB domain (cd01177), highly conserved in all species NF-kB transcription factors such as RelA, RelB, p50 and Relish (Fig. 5B). The XP_002399379 sequence encodes for a Dorsal-Dif (Dorsal-related immunity factor)-like factor probably involved in the tick Toll pathway while the XP_002434504 sequence likely encodes for Relish that may be required for the IMD pathway described in other organisms [21]. These results showed that NF-kB transcription factors are present in *I. scapularis* tick cells with binding site sequences similar to those found in other organisms.

### NF-kB Transcription Factors Bind to *subolesin* Core Promoter

A putative NF-kB-binding site was predicted at positions −279 to −270 on the *subolesin* core promoter with a sequence (GTGTCTTTCC), 80% identical to the consensus NF-kB-binding site (GGGACTTTCC) (Fig. 1B). NF-kB-binding sites (1,742 and 1,912 hits for sequences identical to GGGACTTTCC and GTGTCTTTCC, respectively) were also mapped to *D. melanogaster* chromosome 3L (NT_037436.3) where *akirin* gene is located (3L: 7362900,7366958). Some of these putative NF-kB-binding sites were located at less than 25 kb of the *akirin* gene (Fig. S1).

Electrophoretic mobility shift assays demonstrated that NF-kB transcription factors RelA (p65) and p50 bind to subolesin promoter (Fig. 5C), recognizing the predicted GTGTCTTTCC binding site at positions −279 to −270 (Fig. 1B). Binding activity of p50 was higher than that of p65 (data not shown), reflecting the differences in DNA binding affinity of the NF-kB subunits found in mammals [36].

### NF-kB Transcription Factors are Involved in the Regulation of *subolesin* Gene Expression in Ticks

**Figure 6:**
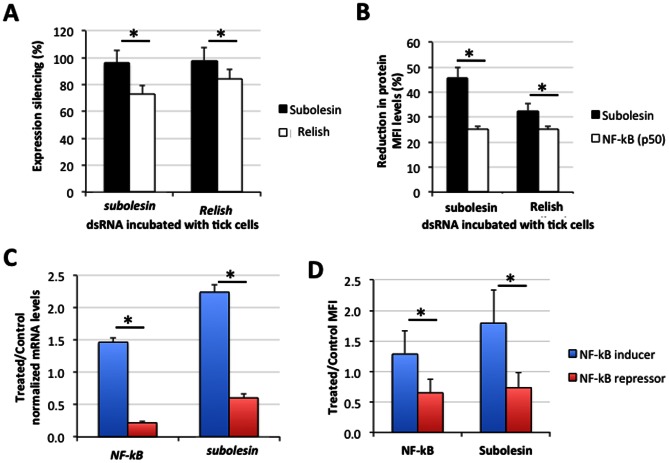
NF-kB transcription factors are involved in the regulation of *subolesin* expression in ISE6 tick cells. (A) For gene knockdown by RNAi, ISE6 tick cells were incubated with *subolesin* or *NF-kB* (*Relish*) dsRNAs and compared to control cells incubated with the unrelated *Rs86* dsRNA. The *subolesin* and *Relish* mRNA levels were determined by real-time RT-PCR and normalized against tick *16S rRNA*. The percent silencing of gene expression was calculated by comparing the normalized Ct values for each treated cell well against the average control normalized Ct value and plotted as average+S.D. (N = 4). The normalized Ct values were compared between treated and control cells by Student’s t-test with unequal variance and were statistically significant (*P≤0.05). (B) Subolesin and NF-kB protein levels were determined after gene knockdown by flow cytometry. MFI for NF-kB and Subolesin was calculated as the MFI of the test-labeled sample minus the MFI of the isotype control and used to calculate expression silencing by comparison to the MFI of the *Rs86* dsRNA-treated control. MFI values (Ave+S.D.; N = 2) were compared between groups by Student’s T-test with unequal variance and were statistically significant (*P≤0.05). (C) Subolesin and NF-kB mRNA levels in ISE6 tick cells after induction and repression of NF-kB. The mRNA levels were determined by RT-PCR and normalized Ct values were calculated for *NF-kB* (*Relish*) and *subolesin*. (D) Subolesin and NF-kB protein levels in ISE6 tick cells after induction and repression of NF-kB. Protein levels were determined by flow cytometry and MFI for NF-kB and Subolesin was calculated as the MFI of the test-labeled sample minus the MFI of the isotype control. For both mRNA and protein levles, Treated/Control ratios (Ave+S.D.; N = 2) were compared between groups by Student’s T-test (*P≤0.05).

RNAi experiments in *I. scapularis* female ticks suggested that NF-kB (Relish) was involved in the regulation of *subolesin* gene expression [3]. In ISE6 tick cells, RNAi experiments did not affect cell viability and showed that *NF-kB* (*Relish*) knockdown resulted in *subolesin* downregulation while *subolesin* knockdown also reduced *Relish* mRNA levels (Fig. 6A). Flow cytometry analysis showed that *subolesin* or *Relish* knockdown by RNAi resulted in lower Subolesin and NF-kB protein levels (Fig. 6B).

The results from *subolesin* and *Relish* gene knockdown in ticks and ISE6 tick cells together with the p65 and p50 binding experiments shown herein in tick cells support the hypothesis that NF-kB participates in the transcription of *subolesin* while Subolesin may be involved in the regulation of *NF-kB* expression [3], [17]. These results agreed with previous experiments in *D. melanogaster* and mice suggesting that Akirins are involved in the regulation of *NF-kB* and other transcription factors [3], [37]–[39].

To further explore this hypothesis, chemical induction and repression of NF-kB was conducted in ISE6 tick cells to characterize Subolesin mRNA and protein levels. The results showed that NF-kB induction and repression did not affect cell viability and were accompanied with Subolesin induction and repression, respectively at the mRNA (Fig. 6C) and protein levels (Fig. 6D). These results strongly suggested that NF-kB transcription factor is involved in the regulation of *subolesin* expression in ticks.

### Subolesin and NF-kB are Involved in Tick Cell Response to Pathogen Infection

**Figure 7:**
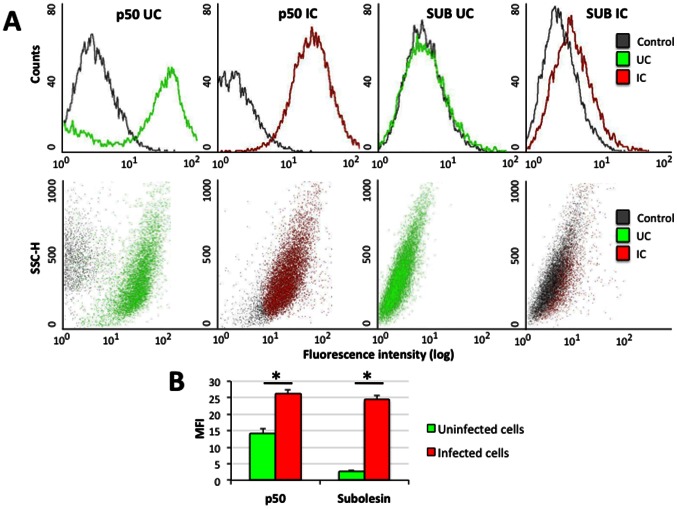
Subolesin and NF-kB protein levels are higher in ISE6 tick cells infected with *A. phagocytophilum*. (A) Flow cytometric profile histogram and plot for the isotype control (grey), NF-kB (p50) and Subolesin (SUB) in uninfected (UC; green) and *A. phagocytophilum*-infected (IC; red) tick cells. (B) MFI for NF-kB and Subolesin in infected and uninfected tick cells. MFI was calculated as the MFI of the test-labeled sample minus the MFI of the isotype control. Results (Ave±S.D.; N = 2) were compared between groups by Student’s T-test (*P≤0.05).

The infection of ISE6 tick cells with *A. phagocytophilum* increased NF-kB RelA (p65) and RelB binding activity (Figs. 5A and 5B) and NF-kB (p50) protein levels (Figs. 7A and 7B) in infected ISE6 tick cells. Furthermore, although as in previous experiments *subolesin* mRNA levels did not change in response to *A. phagocytophilum* infection of ISE6 tick cells [40], [41], protein levels were higher in infected than uninfected cells (Figs. 7A and 7B), probably reflecting regulation at the post-transcriptional level. Recently, the proteome of ISE6 tick cells was characterized in response to *A. phagocytophilum* infection (unpublished results) and showed that one of the significantly increased proteins in infected cells was the ribosomal protein 3A (RP3A; XP_002402816; 3.1-fold increase, false discovery rate (FDR) = 0.004) which has been shown to be an integral subunit of the NF-kB transcription factor [19]. Together, these results showed that NF-kB and Subolesin protein levels increase in ISE6 tick cells in response to *A. phagocytophilum* infection.

**Figure 8:**
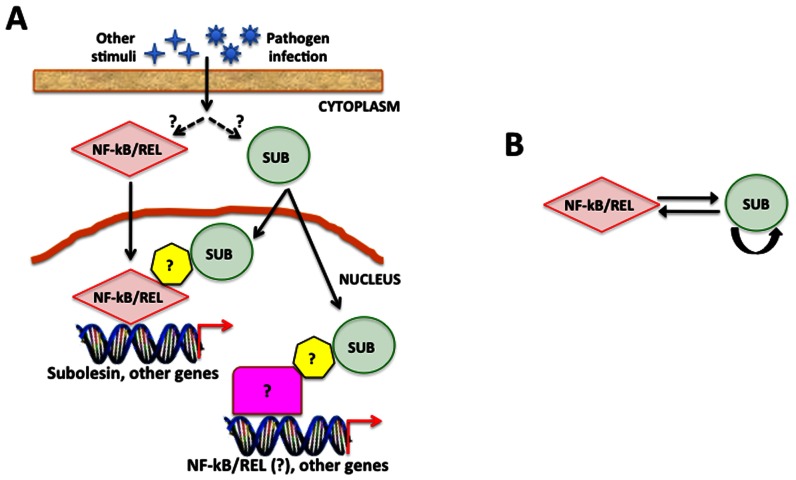
Model for Subolesin regulation in ticks. (A) Proposed model for NF-kB/Relish(REL) and Subolesin (SUB) regulation of gene expression in ticks. The mechanisms resulting in NF-kB/Subolesin activation and/or increased protein levels are still unknown. NF-kB/REL directly regulates the expression of SUB and other genes. SUB interacts with NF-kB/REL subunits through unknown auxiliary proteins and may be involved in the regulation of NF-kB/REL expression and the regulation of NF-kB/REL-independent gene expression in ticks. (B) Proposed regulatory network that includes cross-regulation between NF-kB/REL and Subolesin and Subolesin auto-regulation.

Subolesin/Akirin are functionally conserved and have been proposed to be involved in host innate immune response to pathogen infection that affects the regulation of NF-kB-dependent gene expression [3], [5], [13]–[15], [17], [18], [20], [41]–[43] (Fig. 8A). In addition, Subolesin has a role in other molecular pathways including those required for tissue development and function that are important for pathogen infection and multiplication in ticks [2], [14]. Consequently, a direct effect of *subolesin* knockdown in ticks is reduced innate immunity, thereby increasing pathogen infection levels [44]. Lower pathogen infection levels may result from the effect of *subolesin* knockdown on tissue structure and function and the expression of genes that are important for pathogen infection and multiplication [11], [15], [16]. Collectively, these results support a role for Subolesin and NF-kB transcription factors in tick immune response, but Subolesin is also involved in the regulation of other molecular pathways in ticks.

### Conclusions

Evolutionary and functional studies have shown that *subolesin*/*akirin* genes are conserved and essential to many physiological processes in metazoans [2]–[6], [13], [17], [20] (Fig. 8A). The results presented herein demonstrated that *subolesin*/*akirin* are evolutionary conserved at several levels (primary sequence, gene organization and function), thus showing that these molecules have a crucial biological function in all metazoans. NF-kB regulation of *subolesin* gene expression in ticks suggests that as in other organisms [19], different NF-kB integral subunits and/or unknown interacting proteins regulate the specificity of the NF-kB-mediated gene expression in ticks (Fig. 8A). Additionally, the possible implication of Subolesin in *NF-kB* regulation in ticks adds to the complexity of this regulatory network with important implications in tick immune response (Fig. 8A). Taken together, these results suggest a regulatory network that includes cross-regulation between NF-kB and Subolesin and Subolesin auto-regulation (Fig. 8B). These studies highlight the importance of characterizing gene regulation in ticks, with particular emphasis on immune response pathways and the role of NF-kB and Subolesin in gene regulation to design new strategies for the control of tick infestations and pathogen transmission.

## Materials and Methods

### ISE6 Tick Cells

The ISE6 tick cell line, derived originally from *I. scapularis* embryos (provided by U.G. Munderloh, University of Minnesota, USA) was cultured in L15B medium as described previously [46], [47]. The ISE6 tick cells were inoculated with the NY18 isolate of *A. phagocytophilum* or the Oklahoma isolate of *Anaplasma marginale* and maintained as described previously [31], [40], [41]. Uninfected and infected cultures with approximately 10^7^ cells each were sampled at 13 days post-infection (dpi) with approximately 30% infected cells. Collected cells were centrifuged at 10,000 x g for 3 min and used in various experiments.

### RNA Interference in ISE6 Tick Cells

The dsRNA for *subolesin*, *NF-kB* (*Relish*; XP_002434504) and *Rs86* control were generated with oligonucleotide primers containing T7 promoter sequences for *in vitro* transcription and synthesis of dsRNA using the Access RT-PCR system (Promega, Madison, WI, USA) and the Megascript RNAi kit (Ambion, Austin, TX, USA) as reported previously and using oligonucleotide primers RelRNAi5: 5′-TAATACGACTCACTATAGGGTACTATGTTTCCCTGTAATGTCCG-3′ and RelRNAi3∶5′-TAATACGACTCACTATAGGGTACTGCTCGAGGCAAACTCCCGTC-3′ for *Relish* [2], [3], [48]. RNAi experiments were conducted in cell cultures by incubating ISE6 tick cells with 10 µl dsRNA (5×10^10^–5×10^11^ molecules/µl) and 90 µl L15B medium in 24-well plates using 4 wells per treatment [40]. Control cells were incubated with the unrelated *Rs86* dsRNA. After 48 hours of dsRNA exposure, tick cells were infected with *A. phagocytophilum* (NY18 isolate) or mock infected by adding culture medium alone as described previously [40], [41]. Cells were incubated for an additional 72 hours, collected and used for RNA extraction. RNA was used to analyze *subolesin* and *NF-kB* (*Relish*) mRNA levels by real-time RT-PCR as described previously and using oligonucleotides RelPCR5∶5′-GAGGACTGCTGCTACGTCAC-3′ and RelPCR3∶5′-ACCAGGTTCAGGTTCAGCTC-3′ for *Relish* [45]. Gene knockdown was analyzed by real-time RT-PCR with respect to Rs86 control by comparing the normalized Ct values against tick *16S rRNA* for each treatment against the average control normalized Ct value (N = 4) [3], [41]. The normalized Ct values were compared between treated and control cells by Student’s t-test with unequal variance (P = 0.05).

### Characterization of Subolesin Gene Organization and Sequence

To characterize *subolesin* gene organization and sequence, a cDNA enconding for *I. scapularis subolesin* (IDE8 cells; Genbank accession number AY652654) was aligned with an *I. scapularis* genomic sequence (Wikel tick colony; Genbank accession number DS936446). Total DNA was isolated from *I. scapularis* ISE6 tick cells using TRI Reagent (Sigma, St. Louis, Mo, USA) following manufacturer’s recommendations. Primers were designed to amplify the predicted introns (Table 2) by PCR using the DyNAzyme EXT PCR kit (Finnzymes Oy, Espoo, Finland) in a 50 µl reaction mixture. Total RNA was isolated from ISE6 tick cells and *I. scapularis* tick strains from Oklahoma (OSU; Tick Rearing Facility, Oklahoma State University, Stillwater, OK, USA), New York (NY; D. Sonenshine, Old Dominion University, Norfolk, VA, USA) and Rhode Island (RI1; T.N. Mather, University of Rhode Island, Kingston, RI, USA and RI2; R.F. Massung, Centers for Disease Control and Prevention, Atlanta, GA, USA) using TRI Reagent (Sigma). The *subolesin* cDNAs were amplified by RT-PCR using oligonucleotide primers 4D8R5: (5′-GCTTGCGCAACATTAAAGCGAAC-3′) and 4D8R: (5′-TGCTTGTTTGCAGATGCCCATCA-3′) and the Access RT-PCR system (Promega) as previously described [2]. Control reactions were performed without DNA or RNA to monitor contamination of the PCR reaction. PCR products were electrophoresed in 1% agarose gels to check the size of amplified fragments by comparison to a DNA molecular weight marker (1 Kb Plus DNA Ladder; Promega, Madison, WI, USA). Amplified fragments were purified using a MinElute PCR purification kit (Qiagen, Valencia, CA, USA) and cloned into the pGEM-T vector (Promega) for sequencing both strands by double-stranded dye-determination cycle sequencing and primer walking (Core Sequencing Facility, Department of Biochemistry and Molecular Biology, Noble Research Center, Oklahoma State University, Stillwater, OK, USA). At least three clones were sequenced for each fragment. Sequence alignments were performed between Wikel tick colony and ISE6 tick cells genomic sequences for introns and between Wikel tick colony genomic sequence and ISE6 tick cells and tick strains cDNAs for exons to characterize single-nucleotide polymorphisms (SNPs) and insertion/deletions (INDELs). Sequences were deposited in the Genbank under accession numbers JX898528 (for *subolesin* genomic sequence in ISE6 tick cells) and JX193813-JX193816 (for tick strains cDNAs).

### Mapping of the *subolesin* Transcription Start Site by Automated Capillary Electrophoresis

The analysis of *subolesin* transcription start site was performed using mRNA start site analysis by automated capillary electrophoresis (MSACE; Plant-Microbe Genomics Facility, Ohio State University, OH, USA). MSACE consists on a primer extension assay where the extension product is labeled with a fluorescent dye that is analyzed by automated capillary electrophoresis. RNA from ISE6 cells was isolated using RNeasy mini kit (Qiagen) following manufacturer’s recommendations. The oligonucleotide primer PESUB (5′-ACTATGCAGCGGATCCCAATCG-3′) was labeled with 6FAM (Applied Biosystems, Carlsbad, CA, USA) on the 5′ end and used for the primer extension assay. PESUB is located between positions +27 to +48 after the translation start site of the *subolesin* gene (AY652654). Ten µg RNA were mixed with 100 pmol 6FAM-PESUB primer in a 11 µl total volume. The mix was heated at 90°C for 3 min and then slowly cooled to 30°C in a PCR thermocycler. Primer extension was completed by adding AMV reverse transcriptase (AMV RT) 5X reaction buffer (Promega), 10 mM dNTP mix, 40 U of RNasin ribonuclease inhibitor (Promega) and 25 U of AMV RT enzyme (Promega). The reaction was incubated for 1 h at 42°C. RNA was degraded using 10 ng RNase A (10 µl at 1 ng/µl) for 30 min at 37°C [49]. The resulting cDNA was purified with the MinElute PCR purification kit (Qiagen) and eluted in 15 µl water. The template necessary for the DNA sequencing reaction (520 bp) included 469 bases upstream of the translation start site. The DNA template was amplified with 56PE (5′-GACAGAATCCGACACCCAGA-3′) and PESUB primers (5′-ACTATGCAGCGGATCCCAATCG-3′) by PCR using GoTaq Flexi DNA polymerase (Promega). The specific PCR conditions were 95°C for 2 min followed of 35 cycles of denaturation at 95°C for 30 sec, annealing at 60°C for 30 sec and extension at 72°C for 1 min. The PCR product was purified with the MinElute PCR purification kit (Qiagen) and cloned into the pGEM-T vector (Promega). The extension product was analyzed on a 3730 DNA Analyzer (Applied Biosystems). Results from MSACE analysis were corroborated by 5’ System for Rapid Amplification of cDNA Ends (RACE; Life Technologies S.A., Madrid, Spain) using gene-specific primers GSP1 (4D8R: 5′-TGCTTGTTTGCAGATGCCCATCA-3′) and GSP2 (3PRO: 5′-CCGGATCCGCTCCGGATGATGAACTTTT-3) and following manufacturer’s recommendations [50]. The PCR product was purified, cloned into pGEM-T and 20 clones were sequenced as previously described.

### Characterization of *subolesin* Core Promoter

A 5,503 pb region was used to characterize *subolesin* promoter sequence. DNA was isolated from ISE6 tick cells as previously described. Oligonucleotide primers were designed to amplify by PCR 7 different fragments with 5′ or 3′ deletions in the *subolesin* promoter region (Table 3). PCR reactions were performed using the Advantage Genomic LA Polymerase Mix (Clontech, Mountain View, CA, USA) in a 25 µl reaction mixture. Control reactions were performed without DNA to monitor contamination of the PCR reaction. PCR products were electrophoresed, purified, cloned into pGEM-T and sequenced as previously described. Positives clones from the seven fragments were digested with *BamH*I and *Bgl*II (Promega), purified using the Qiaquick Gel Extraction kit (Qiagen) and cloned into the pDsRed-Express-1 vector (Clontech), which is a promoterless vector containing the *Discosoma* sp. red fluorescent protein (DsRed) marker. Resulting constructs were purified and 4 µg were used to transfect ISE6 tick cells using Effectene Transfection Reagent (Qiagen). Two independent experiments were conducted. The pDsRed2-ER expressing the DsRed protein and promoterless pDsRed-Express-1 were used as positive and negative controls, respectively. After transfection, cells were incubated in L15B medium for 24 h [51] and then analyzed for DsRed protein expression by flow cytometry FACS analysis using a FACSCalibur cytometer (Becton Dickinson, Franklin Lakes, NJ, USA).

**Table 3:** Primers and PCR conditions used for *subolesin* promoter analysis.

Promoter fragment	Length (bp)	Upstream/downstream primer sequence (5′-3′)	PCR conditions (annealing, extension)
51	5503	51PRO:GGAGATCTAATGCCGTCAATAGTGCCTT 3PRO: CCGGATCCGCTCCGGATGATGAACTTTT	57°C, 30 s 72°C, 5 min
52	4584	52PRO:CCAGATCTACAAAAGGTTCTCCTGACCC 3PRO: CCGGATCCGCTCCGGATGATGAACTTTT	56°C, 30 s 72°C, 3 min
53	3592	53PRO:CCAGATCTTTTGAGGGAAGTGCTAGAGCG 3PRO: CCGGATCCGCTCCGGATGATGAACTTTT	61°C, 30 s 72°C, 4 min
54	2571	54PRO: CCAGATCTAAAGCGTAGGTTTTCTGCCA 3PRO: CCGGATCCGCTCCGGATGATGAACTTTT	56°C, 30 s 72°C, 3 min
55	1673	55PRO: GGAGATCTAGTCCGCTTTTATCCTTGAAA 3PRO: CCGGATCCGCTCCGGATGATGAACTTTT	56°C, 30 s 72°C, 1 min
56	763	56PRO: CCAGATCTTCAGCCATTTCAAAGTTGCG 3PRO: CCGGATCCGCTCCGGATGATGAACTTTT	56°C, 30 s 72°C, 1 min
SIN56	4734	51PRO: GAGATCTAATGCCGTCAATAGTGCCTT SIN56: CAAACTACGCGCTGTATAAATATCCCC	56°C, 30 s 72°C, 3 min

To confirm these results, the construct containing the identified *subolesin* core promoter (fragment 56) was more efficiently transfected into *Aedes aegypti* mosquito Aag2 cells (kindly provided by Alain Kohl and Claudia Rueckert; [52]). As in the experiment with tick cells, pDsRed2-ER expressing the DsRed protein and promoterless pDsRed-Express-1 were used as positive and negative controls, respectively. Cells were grown at 31°C in L15 medium containing 10% tryptose phosphate broth (Sigma) and 10% fetal bovine serum. Cells were seeded for transfection 24 h prior to use in 24-well plates. Approximately 2x10^5^ cells/well were transfected with 100 µl/well of Lipofectamine 2000 (Invitrogen, Oregon, USA; 1 µl/well) and plasmid DNA (4 µg/well) in Optimem added to 400 µl of medium/well and incubated for 5 h at 31°C following manufacturer’s instructions. After transfection, cells were incubated in L15 medium for 12 and 24 h and collected for FACS as described before. Cells were also observed under a Nikon Eclipse Ti-U fluorescent microscope with a Nikon Digital Sight DS Vi1 camera. Two independent experiments were conducted with similar results.

### DNA-binding ELISA for the Characterization of NF-kB Binding Activity in Tick Cells

To characterize NF-kB binding activity in ISE6 tick cells, a DNA-binding ELISA was used with RelA (p65) and RelB antibodies (Trans AM NF-kB Family Transcription Factor Assay Kit, Active Motif, Carlsbad, CA, USA). ISE6 tick cells were collected in PBS and lysed following manufacturer’s instructions. After centrifugation, the pellet contained the nuclear fraction at a concentration of 4 mg/ml. The wild-type consensus NF-kB oligonucleotide (5′-GGGACTTTCC-3′) was used (20 pmol/well) as a competitor for NF-kB binding in order to monitor the specificity of the assay. Conversely, the mutated consensus oligonucleotide (Active Motif) should have no effect on NF-kB binding and served as negative control. The Raji cells nuclear extract (Active Motif) was used as positive control for NF-kB binding. Four replicates were done for each assay and results were compared between groups by Student’s T-test (P = 0.05).

### Electrophoretic Mobility Shift Assay for the Characterization of NF-kB Binding to *subolesin* Promoter

NF-kB binding to *subolesin* promoter was assessed by incubating 5′ end biotin-labeled double stranded (ds)DNA oligonucleotide corresponding to the surrounding region of the predicted NF-kB binding site in the *subolesin* promoter (nucleotides −309 to −252; Fig. 1B) with p50 and Rel (p65) proteins (Active Motif, Carlsbad, CA USA) according to the manufacturer’s protocol (Gel Shift Chemiluminiscent EMSA kit, Active Motif). NF-kB proteins (0.1 µg) were incubated independently with 20 fmol of labeled dsDNA probe in binding buffer (EMSA kit, Active Motif) in a final volume of 20 µl at room temperature for 20 min. The resulting complexes were separated in a 6% DNA retardation gel (Invitrogen, Madrid, Spain). Competition with the unlabeled probe containing the predicted subolesin NF-kB binding site (GTGTCTTTCC) were done in a 200∶1 (unlabeled:labeled) ratio.

### Characterization of Subolesin and NF-kB Expression in Tick Cells

Subolesin and NF-kB expression was characterized in ISE6 tick cells in response to *A. phagocytophilum* infection, after gene knockdown by RNAi and after activation and repression of the NF-kB pathway. Tick cells were infected with *A. phagocytophilum* or left uninfected as described before. For the activation and repression of the NF-kB pathway, ISE6 tick cells were incubated at 31°C for 24 hrs with 7.5 mg/ml NF-kB inducer (2-Deoxy-D-glucose; Sigma, St. Louis, MO, USA) [53], 5 mM NF-kB represor (Sodium Salicylate; Santa Cruz Biotechnology, Inc., Santa Cruz, CA, USA) [54], and PBS as control and analized by RT-PCR and flow cytometry for subolesin and NF-kB (Relish or p50) expression.

The *subolesin* and *NF-kB* (*Relish*) mRNA levels were characterized by real-time RT-PCR as described before for RNAi. For flow cytometry analysis, ISE6 tick cells were washed in phosphate buffered saline (10 mM PBS) fixed and permeabilized with Intracell® fixation and permeabilization kit (Inmunostep, Salamanca, Spain) following manufacturer recomendations. After permeabilization, the cells were washed in PBS and incubated in 100 µl with primary unlabeled rabbit polyclonal antibodies (preimmune IgG isotype control; 5 µg/ml, anti-subolesin; 5 µg/ml) and labelled monoclonal antibodies (PE-anti-NF-kB p50; 40 µg/ml), washed in PBS and incubated in 100 µl of PBS with FITC or Cy5-goat anti-rabbit IgG for subolesin (Sigma, Madrid, Spain) labeled antibodies (diluted 1/500) for 15 min at 4°C. Finally, the cells were washed with PBS and resuspended in 500 µl of PBS. All samples were analyzed on a FACScalibur® Flow Cytometer, equipped with the CellQuest Pro® software (BD-Biosciences, Madrid, Spain). The viable cell population was gated according to forward scatter and side scatter parameters. The level of Subolesin and NF-kB in the viable cells was determined as the geometric median fluorescence intensity (MFI) of the test-labeled sample minus the MFI of the isotype control [55]. Two replicates were done for each assay and results were compared between groups by Student’s T-test (P = 0.05).

### Sequence Analysis

Multiple sequence alignments were performed using Geneious Pro 5.0.3 software (Biomatters Ltd., Auckland, New Zealand; http://www.geneious.com). Transcription factor binding sites to subolesin promoter were predicted using TRANSFAC at Biobase (http://www.biobase-international.com/product/transcription-factor-binding-sites). Blasting against nonredundant sequence database (nr) and databases of tick-specific (http://www.ncbi.nlm.nih.gov and http://www.vectorbase.org/index.php) and *D. melanogaster* release 5.30 genomic sequences was done using tblastx and blastn (http://blast.ncbi.nlm.nih.gov/Blast.cgi). Protein sequences were aligned using the CLUSTAL 2.1 multiple sequence alignment tool (EMBL-EBI; http://www.ebi.ac.uk/Tools/). Conserved protein domains (cd) were analyzed using Conserved Domains and Protein Classification at ncbi (http://www.ncbi.nlm.nih.gov/Structure/cdd/cdd.shtml) [56]. *D. melanogaster* akirin and NF-kB-binding sites were mapped using the NCBI Map Viewer (http://www.ncbi.nlm.nih.gov/mapview/).

## Supporting Information

Figure S1 NF-kB-binding sites in *D. melanogaster* chromosome 3L (NT_037436.3) where akirin gene is located (3L: 7362900,7366958). Blasting was done against GTGTCTTTCC sequence in *D. melanogaster* release 5.30 genomic sequence using blastn (http://blast.ncbi.nlm.nih.gov/Blast.cgi) and mapped using the NCBI Map Viewer (http://www.ncbi.nlm.nih.gov/mapview/).
(TIF)
