# Supplementary figures and images for: Reciprocal Regulation of NF-kB (Relish) and Subolesin in the Tick Vector, Ixodes scapularis
Source: PLoS One. 2013 Jun 12;8(6):e65915. doi: 10.1371/journal.pone.0065915 (PMC3680474; doi:10.1371/journal.pone.0065915)

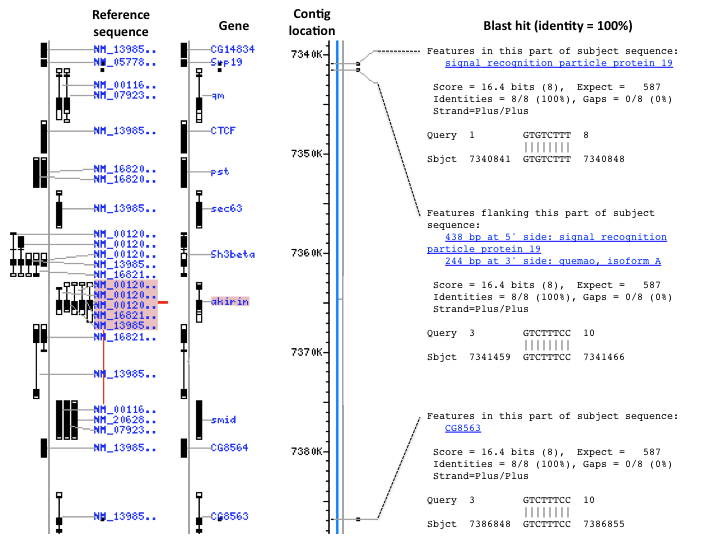

Supplement: Figure S1 — NF-kB-binding sites in D. melanogaster chromosome 3L (NT_037436.3) where akirin gene is located (3L: 7362900,7366958). Blasting was done against GTGTCTTTCC sequence in D. melanogaster release 5.30 genomic sequence using blastn (http://blast.ncbi.nlm.nih.gov/Blast.cgi) and mapped using the NCBI Map Viewer (http://www.ncbi.nlm.nih.gov/mapview/). (TIF) [file pone.0065915.s001.tif]
